# Studies on the Biodiversity of Halophilic Microorganisms Isolated from El-Djerid Salt Lake (Tunisia) under Aerobic Conditions

**DOI:** 10.1155/2009/731786

**Published:** 2009-11-30

**Authors:** Abdeljabbar Hedi, Najla Sadfi, Marie-Laure Fardeau, Hanene Rebib, Jean-Luc Cayol, Bernard Ollivier, Abdellatif Boudabous

**Affiliations:** ^1^Laboratoire Microorganismes et Biomolécules Actives, Faculté des Sciences de Tunis, Université de Tunis El Manar, 2092 Tunis, Tunisia; ^2^Laboratoire de Microbiologie et de Biotechnologie des Environnements Chauds, UMR180, IRD, Universités de Provence et de la Méditerranée, ESIL case 925, 13288 Marseille cedex 9, France

## Abstract

Bacterial and archaeal aerobic communities were recovered from sediments from the shallow El-Djerid salt lake in Tunisia, and their salinity gradient distribution was established. Six samples for physicochemical and microbiological analyses were obtained from 6 saline sites in the lake for physico-chemical and microbiological analyses. All samples studied were considered hypersaline with NaCl concentration ranging from 150 to 260 g/L. A specific halophilic microbial community was recovered from each site, and characterization of isolated microorganisms was performed via both phenotypic and phylogenetic approaches. Only one extreme halophilic organism, domain *Archaea*, was isolated from site 4 only, whereas organisms in the domain *Bacteria* were recovered from the five remaining sampling sites that contained up to 250 g/L NaCl. Members of the domain *Bacteria* belonged to genera *Salicola, Pontibacillus, Halomonas, Marinococcus*, and *Halobacillus*, whereas the only member of domain *Archaea* isolated belonged to the genus *Halorubrum*. The results of this study are discussed in terms of the ecological significance of these microorganisms in the breakdown of organic matter in Lake El-Djerid and their potential for industry applications.

## 1. Introduction

Hypersaline environments are found in a wide variety of aquatic and terrestrial ecosystems. They are inhabited by halotolerant microorganisms but also halophilic microorganisms ranging from moderate halophiles with higher growth rates in media containing between 0.5 M and 2.5 M NaCl to extreme halophiles with higher growth rates in media containing over 2.5 M NaCl [1]. Aerobic, anaerobic, and facultative anaerobic microbes belonging to domains *Archaea *and *Bacteria* have been recovered from these extreme ecosystems, where they participate in overall organic matter oxidation [2–6].

Moderate and extreme halophiles have been isolated not only from hypersaline ecosystems (salt lakes, marine salterns and saline soils) but also from alkaline ecosystems (alkaline lakes). The most widely studied ecosystems are the Great Salt Lake (Utah, USA), the Dead Sea (Israël), the alkaline brines of Wadi Natrun (Egypt), and Lake Magadi (Kenya) [7–9]. It is noteworthy that low taxonomic biodiversity is observed in all these saline environments [10, 11], most probably due to the highly salt concentrations measured in these environments. 

To adapt to high saline conditions, halophilic microorganisms have developed various biochemical strategies, including compatible solute synthesis to maintain cell structure and function [12–14]. These solutes (e.g., ectoïne) plus other compounds (bacteriorhodopsins, exopolysaccharides, hydrolases, biosurfactants) produced by halophilic microbes are clearly of industrial interest. Besides these metabolical and physiological features, halophilic microorganisms are known to play important roles in fermenting fish sauces and in transforming and degrading waste and organic pollutants in saline wastewaters [15–17].

Southern Tunisia features numerous ecosystems including extreme (hypersaline) environments in which microbial diversity has been poorly studied. El-Djerid Sebkha, the largest saline lake (5000 km^2^) in southern Tunisia, is an important source of salt for food, but its microbial diversity has never yet been studied. Given its economic value for the region as a salt source, we conducted a microbial survey to gain better knowledge of the microbial diversity thriving in this extreme ecosystem. The purpose of this research was to chemically analyse salt and brine samples collected from the lake, isolate any novel extremely halophilic aerobic or facultative anaerobic microorganisms, and examine their phenotypic features and physiological and biochemical characteristics with a view to screening for metabolites of industrial interest produced by the novel halophilic isolates.

## 2. Material and Methods

### 2.1. Sample Collections

**Figure 1:**
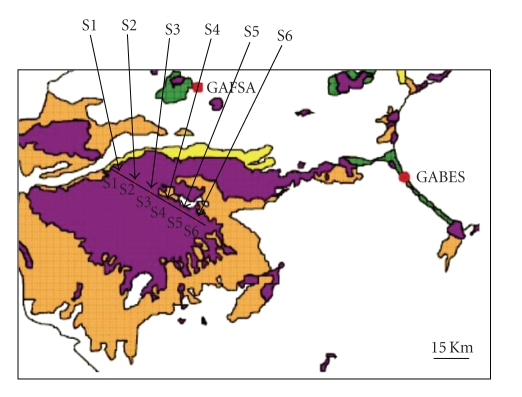
Site map of the El-Djerid lake (Tunisia) and sampling points.

The studied strains were isolated from water and sediments of the El-Djerid Sebkha, a shallow lake located in southern Tunisia. According to in situ physico-chemical conditions and level of wastewater pollutants, the Sebkha was divided into six experimental sites (Figure 1). The samples were collected in February 2006. Water and sediment samples were collected at the surface and at various depths (0.1, 0.2, 0.3 m) in each site. All samples were collected into sterile bottles and stored in ice boxes in the laboratory.

### 2.2. Physicochemical Analysis of the Samples

pH, moisture content, and Na^+^, K^+^, Ca^2+^, Mg^2+^, and Cl^−^ content of the salt and sediment samples were measured according to standard methods of Trussel et al. [18]; Cl^−^ was quantified by titration with AgNO_3_, Mg^2+^ was quantified by atomic absorption spectrophotometry, Na^+^ was quantified by flame spectrophotometry, and Ca^2+^ was quantified by complexometry using EDTA. Temperature and pH were measured in situ.

### 2.3. Enrichment and Isolation

Enrichment cultures and isolation procedures to recover aerobic or facultatively anaerobic moderately to extremely halophilic microorganisms were performed in medium containing (per liter): NaCl, 250 g; MgCl_2_ 6H_2_O, 13 g; MgSO_4_ 7H_2_O, 20 g; KCl, 4 g; CaCl_2_ 2H_2_O, 1 g; NaBr, 0.5 g; NaHCO_3_, 0.2 g; yeast extract, 5 g; tryptone, 8 g; and glucose, 1 g. pH was adjusted to 7.2 with 10 M NaOH before autoclaving. Enrichment cultures were subcultured several times under the same conditions. Strains were grown in 100 mL of medium in 250-mL Erlenmeyer flasks in a rotary shaker at 37°C under agitation at 150 rpm. Aliquots (100 *μ*l) of 10^−1^–10^−4^ dilutions were plated onto agar medium. After two weeks of incubation at 37°C, there were red, orange-red, pale-pink, yellowish, cream, white, and transparent colonies. Different colonies were picked and restreaked several times to obtain pure cultures. Microbial cultures were stored at −80°C in the isolation medium supplemented with 50% glycerol.

### 2.4. Characterization and Identification of Isolates

Among the 130 strains isolated, only 36 showed different phenotypic characteristics and phylogenetic signatures (ARDRA, 16S rRNA gene sequences). These were chosen for further characterization. Isolates were examined for colony and cell morphology and motility. Colonial morphologies were described using standard microbiological criteria, with special emphasis on pigmentation, diameter, colonial elevation, consistency, and opacity [19]. These characteristics were described for cultures grown at optimum temperature, pH, and salt concentration.

For biochemical tests, the strains were grown in flasks and cultures were incubated at 37°C. The optimal ionic content (per liter: 4 g of KCl, 13 g of MgCl_2_ 6H_2_O, 1 g of CaCl_2_ 2H_2_O, 20 g of MgSO_4_ 7H_2_O, 0.5 g of NaBr, 0.2 g of NaHCO_3_, 250 g of NaCl) was used in all the biochemical test media. Oxidase reaction was performed according to Kovacs (1956) [20]. Catalase was determined by adding 10 volumes of H_2_O_2_ to each strain culture (after 18 hour incubation at 37°C) on solid medium. Gelatinase, *β*-galactosidase, urease, indol production, and Voges-Proskauer tests were performed using standard procedures. Other phenotypic characteristics were determined using API 20E and API 20NE kits (BioMérieux, Marcy l'Etoile, France) according to Logan and Berkeley (1984) [21].

### 2.5. PCR Amplification of 16S rDNA

The DNA from bacterial cultures was extracted using a Wizard Genomic DNA Purification Kit. The 16S rRNA gene of the isolate strain was amplified by adding 1 *μ*L of cell culture to a thermocycler microtube containing 5 *μ*L of 10 × taq buffer, 0.5 *μ*L of each 50 nM Fd1 and Rd1 primers, 5 *μ*L of 25 mM MgCl_2_ 6H_2_O, 0.5 *μ*L of 25 mM dNTPs, 0.5 *μ*L of Taq polymerase (5U *μ*L^−1^), and 38 *μ*L of sterilized distilled water. Universal primers Fd1 and Rd1 (Fd1, 5′-AGAGTTTGATCCTGGCTCAG-3′ and Rd1, 5′-AAGGAGGTGATCCAGCC-3′) were used to obtain a PCR product of ~1.5 kb corresponding to base positions 8-1542 based on *Escherichia coli *numbering of the 16S rRNA gene [22]. The sample was placed in a hybrid thermal reactor thermocycler (BIOMetra, Leusden, The Netherlands), denatured for 1 minute at 95°C and subjected to 30 cycles for 20 seconds at 95°C, 30 seconds at 55°C, and 1 minute and 30 seconds at 72°C. This was followed by a final elongation step for 5 minutes at 72°C. The PCR products were analysed on 1% (w/v) agarose gels and sent to GATC (Germany) for sequencing. Sequence data were imported into the BioEdit version 5.0.9 sequence editor [23]; base-calling was examined, and a contiguous sequence was obtained. The full sequence was aligned using the RDP Sequence Aligner program [24]. The consensus sequence was manually adjusted to conform to the 16S rRNA gene secondary structure model [22]. A nonredundant BLAST search [25] identified its closest relatives. Sequences used in the phylogenetic analysis were obtained from the RDP [24] and GenBank databases [26]. Sequence positions and alignment ambiguities were eliminated and pairwise evolutionary distances were calculated using the method of Jukes and Cantor (1969) [27]. A dendrogram was constructed using the neighbour-joining method [28]. Confidence in tree topology was determined using 100-bootstrapped trees [29].

### 2.6. Restriction Endonuclease Digestions

Enzymatic digestions were performed by incubating 5 *μ*L of the PCR products with 10 U of each endonuclease and the corresponding enzyme buffer. Digestions were continued for one hour at 37°C for *Alu*I, *Hae*III, and *Rsa*I. Digested products were analysed on 2% (w/v) agarose gels.

## 3. Results

### 3.1. Physicochemical Analyses

Temperature at the sampling sites was 15°C at 6 A.M. The physico-chemical characteristics of the sediment samples are shown in Table 1. The pH of sediment samples was between 7.8 and 8.8. The highest moisture content values were found in the S4 sample. Na^+^  content was the highest in the S5 sample, and Ca^2+^ content was the highest in the S3 sample, whereas K^+^ concentration was the highest in the S2 sample (Table 1). Total salt composition was higher at the S4 sampling site (335 mg/g) than the other sampling sites (Table 1). All sediment samples from the studied lake were dominated by Cl^−^ and high levels of Ca^2+^. Total ionic composition of the lake differed depending on the area sampled. Given the mineral composition of the lake and its concentration in Na^+^, K^+^, Ca^2+^, Mg^2+^, and Cl^−^, it should clearly be inhabited by halophilic microorganisms, thus justifying the microbial survey.

### 3.2. Microbiological Analyses

**Figure 2:**
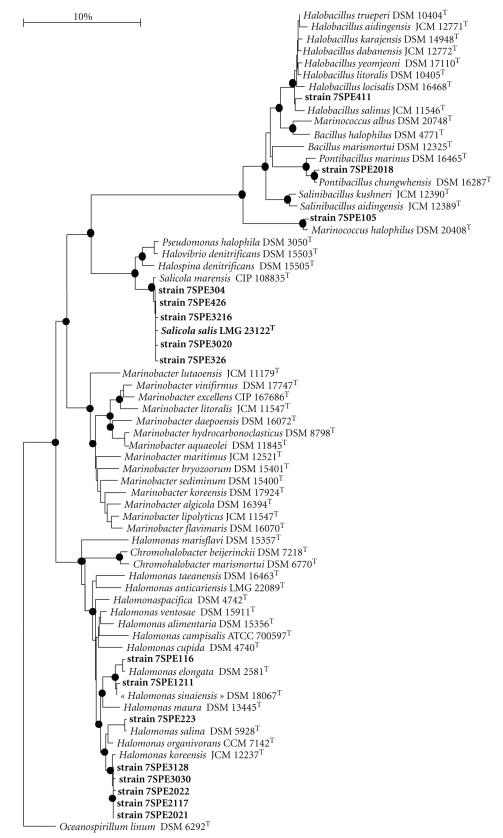
16S rRNA gene-based phylogenetic tree of the bacterial domain, including 16S rDNA sequences from sediment samples from El-Djerid lake. Topologies of the phylogenetic tree built using maximum-likelihood and maximum-parsimony algorithms were similar to those of the tree constructed by neighbour-joining analysis. Solid circles indicate nodes with a bootstrap value higher than 80%.

After several dilutions and subculturing in the same liquid medium under aerobic conditions, colonies were isolated in the agar medium containing 25% NaCl. A total of 130 extremely halophilic strains were isolated under aerobic conditions from the six samples. However, on the basis of phenotypic characteristics (macro and microscopic analysis), physiological analyses (NaCl, pH), biochemical tests (API 20E, API 20NE), and molecular approaches [PCR 16S, ARDRA (digestion by three enzymes *Alu*I, *Hae*III and *Rsa*I)], only 36 isolates were selected and examined in greater detail. These strains were identified by analyzing sequences of genes encoding for 16S rRNA (Figure 2). The highest total bacterial number (1 × 10^4^ cfu/g) growing under aerobic conditions was found in the S1 sampling site. Some colonies were white or transparent whereas others showed various pigmentations, that is, red, orange-red, bright-pink, or yellowish-cream. Cream-coloured colonies were found to be the most numerous in the lake (Table 3).

### 3.3. Colony and Cell Morphology

The dominant bacterial population comprised motile or nonmotile, gram-positive microorganisms, most of which were spore-forming bacteria. Most colonies on Brown agar medium were 0.5–2 mm in diameter after 3 weeks of incubation. These colonies were smooth, circular, low-convex, transparent or translucent, and entire. Cells of all strains isolated were short, long, and swollen rods that occurred in singles, pairs, or short chains. The cells were approximately 0.5–2 *μ*m wide and 2.5–6.5 *μ*m long. The isolates were facultatively anaerobic or aerobic and required yeast extract for growth. The characteristics of all strains are shown in Table 3. Colony pigmentation from these samples ranged from blood-red to pale-pink. Most colonies were 1 to 2 mm in diameter, circular, convex, glistening, and entire. Optimum growth occurred at 25% (w/v) NaCl, 37°C, and pH 7.3, thus suggesting that these isolates should be considered as extremely halophilic according to the definition of Ventosa et al. [1].

### 3.4. Biochemical Tests

Selected strains were tested in biochemical test media (Table 3). ONPG and gelatin hydrolysis were found to be negative and tryptophan deaminase was not produced, whereas lysine decarboxylase, ornithine decarboxylase, and arginine dihydrolase were found to be positive for the majority of isolates. Most of the strains reduced nitrate to nitrite (Table 3).

### 3.5. Phylogenetic Analysis

Based on the enzymatic digestion profiles obtained, 16 representative bacteria of the 36 isolates were chosen for taxonomic and phylogenetic studies. To determine their phylogenetic position, the 16S rRNA gene sequence of each strain was analyzed, and a phylogenetic tree was constructed based on 1280 unambiguous bp (Figure 2). The 16S rRNA gene sequences of the strains have been deposited in the GenBank database 

Phylogenetic analysis indicated that the majority of strains isolated are related to genera *Halomonas* or *Salicola*, whereas the other strains are most closely related to species of genera *Halobacillus*, *Pontibacillus,* and *Marinococcus *(Figure 2). All strains shared more than 97% identity with their closest phylogenetic relative (Table 2), suggesting that they should be considered at the same species level until the results of DNA/DNA hybridization studies can validate their affiliation (work in progress). Only one strain representative of the domain *Archaea* was identified as *Halorubrum* sp., but this microorganism was not further characterized. The 16S rRNA gene sequences recovered from the 16 representative isolated bacteria made up 9 taxonomically distinct microorganisms whose closest phylogenetic relatives are *Halobacillus salinus*, *Pontibacillus chungwhensis*, *Marinococcus halophilus*, *Salicola marensis*, *S. salis*, *Halomonas elongata*, *H. sinaiensis*, *H. salina,* and *H. koreensis*.

## 4. Discussion

Recent decades have seen a surge in studies on extreme environments including hypersaline ecosystems. Both molecular and microbiological studies have revealed the presence of moderately to extremely halophilic microorganisms in a wide range of these saline environments [9, 30–33]. El-Djerid salt lake is a hypersaline environment in southern Tunisia that is considered athalassohaline because its salt composition derives from the dissolution of minerals of continental origin [34]. Similarly to other hypersaline ecosystems, the lake is subjected to drastic physico-chemical conditions including high salinity, high radiation (UV) and strong changes in temperatures and dryness which make it a relevant study target for microbiologists. To our knowledge, this is the first microbiological study on extremely halophilic aerobic bacteria from El-Djerid salt lake.

Table 1 reports the results of physico-chemical analysis of soil samples from the six sites. The samples differ from those of the other hypersaline environments studied so far. Sodium and potassium concentrations are higher at the six sites than in the Dead Sea in Israël [8]. In contrast to the waters of the Dead Sea and the Great Salt Lake in the USA, which are slightly acidic (pH 6 to 7), the pH of sites 5 and 6 is 8.3 and 8.2, respectively, and should therefore be considered weakly alkaline. The pH of Lakes Wadi Natrun and Magadi (in Kenya) is considered as highly alkaline environments (pH 11) [35, 36]. 

Throughout the course of this work, we isolated 130 extremely halophilic strains and further characterized 36 of these strains showing different pigmentations with colonies on agar plates (Table 2). Phylogenetic analysis indicated that all isolates were members of five genera of the domain *Bacteria*, including *Salicola*, *Pontibacillus*, *Halomonas*, *Marinococcus,* and *Halobacillus*. Members of the genera *Salicola*, *Pontibacillus, Marinococcus, *and* Halobacillus *are considered aerobic microorganisms, whereas members of genus *Halomonas* are considered facultative anaerobes able to use nitrate as terminal electron acceptor under anaerobic conditions. All these microorganisms may use various organic compounds including sugars as substrates and should be considered chemoorganotrophs. Almost all these isolates were detected on the surface of sediments as well in the first centimetres down (0.1-0.2 m) of each biotope. *Halomonas* species were distributed in all 5 sites studied and represented the major strains isolated, especially in site 1. Members of this genus together with those of the genera *Salicola*, *Pontibacillus*, *Marinococcus*, and *Halobacillus* have also been isolated from other saline environments, including athalassohaline and thalassohaline lakes and marine waters [37–40]. It should be underlined that among the halophilic microbes isolated, only one, which originated from site 4, belonged to the domain *Archaea* (data not shown). However, the limited number of halophilic archaeons detected in El-Djerid lake may be due to the culture media used, which may have favoured bacterial growth and thus do not reflect their real distribution within the lake. All bacterial strains were found as gram-positive rods producing lysine decarboxylase, ornithine decarboxylase, and arginine dihydrolase. Some of the isolates are able also to reduce nitrate to nitrite, suggesting that they may be involved in the global nitrogen cycle within the lake. Since all the isolates are able to grow optimally in the presence of 25% NaCl, they should be considered extremely halophilic [41] and therefore of ecological significance with regard to the biogeochemistry of the El-Djedid Lake in its most saline parts. Surprisingly, despite the fact that site 5 had suitable physico-chemical conditions to allow microbial life, no isolate was recovered from it. 

Extensive research on different hypersaline habitats in Spain and Morocco that focused on the screening of new exopolysaccharide-(EPS)-producing bacteria resulted in several strains isolated from saline soils and described as new species belonging to the genus *Halomonas* [42–46]. Similarly to the observations reported here, a minority of these isolated microorganisms were identified as members of genera *Pontibacillus*, *Marinococcus,* and *Halobacillus*. Several other aerobic or facultatively anaerobic, moderately halophilic bacteria have been classified within genera related to the order* Bacillales *[47]. The potential industrial use of these microorganisms has been underlined (production of compatible solutes, biopolymers, and bioremediation processes) and reviewed in detail [1, 48, 49], prompting us to screen our collection of halophiles for molecules of industrial interest (work in progress). 

Finally, the metabolic features of the extremely halophilic isolates from the El-Djerid salt lake indicated that most of these isolates were able to oxidize organic polymers in the Sebkha and should therefore participate in the mineralization of resident organic matter, similarly to other hypersaline ecosystems [37]. Studies on these bacteria should be reinvestigated as they constitute a source of halostable enzymes (Table 3) that offer potential applications in various pharmacochemical industries [50, 51].

## Figures and Tables

**Table 1:** Physico-chemical characteristics of the sediment samples.

Sampling site	Colour of sampling site	pH	Hardness (%)	Ca^2+^ (mg/g)	Mg^2+^ (mg/g)	Cl^−^ (mg/g)	Na^+^ (mg/g)	K^+^ (mg/g)	Total
S1	Dark-cream	7.9	7.54	80.52	19.42	131.50	26.82	8.55	266.83
S2	Cream	7.5	4.22	68.28	21.37	177.96	44.62	14.40	326.65
S3	Cream	7.9	6.92	82.43	15.84	113.66	69.22	7.10	288.27
S4	Cream	7.3	7.78	57.94	12.26	190.50	67.14	7.48	335.34
S5	Dark-cream	8.3	6.60	64.36	14.94	110.93	76.47	10.42	277.14
S6	Brown-black	8.2	7.29	75.57	20.48	141.60	59.41	10.45	307.54

Average		7.87	6.72	71.52	17.39	144.36	57.28	9.73	300.29

**Table 2:** Distribution and taxonomic characteristics of bacteria and archaea isolated from the 6 sampling sites in El-Djerid Lake.

Organisms	Number of strains/site						
	S1	S2	S3	S4	S5	S6	Number of strains
*Halomonas* sp.	10	8	2	3	0	3	26
*Salicola* sp.	0	0	5	1	0	0	6
*Pontibacillus* sp.	0	1	0	0	0	0	1
*Marinococcus* sp.	1	0	0	0	0	0	1
*Halobacillus* sp.	0	0	0	1	0	0	1
Others (Archaea)^(a)^	0	0	0	1	0	0	1
Total strains	11	9	7	6	0	3	36

^(a)^data not shown.

**Table 3:** Phenotypic features of the 36 strains studied.

	Strains (7SPE)							
Sampling sites (S)/depth (cm)/ characteristics	2021 S2/0	2117 S2/10	2022 S2/0	3′030S3/0	3′128S3/10	223 S2/20	1′211S1/20	116 S1/10

Taxonomical status	*Halomonas* sp.	*Halomonas* sp.	*Halomonas* sp.	*Halomonas* sp.	*Halomonas* sp.	*Halomonas* sp.	*Halomonas* sp.	*Halomonas* sp.
Colonial morphology	Circular	Circular	Circular	Circular	Circular	Circular	irregular and spreading	irregular and spreading
Colony size	1 mm	1-2 mm	1 mm	1-2 mm	1 mm	1-2 mm	2 mm	1-2 mm
Colony	convex	convex	convex	slightly raised	flat	convex	convex	convex
Colony density	opaque matt	opaque matt	opaque matt	translucent glistening	opaque matt	opaque matt	translucent glistening	translucent glistening
Pigmentation	cream	white	cream	transparent-white	cream	cream	white	white
Cell shape	pleomorphic rods	pleomorphic rods	pleomorphic cells	pleomorphic cells	pleomorphic rods	pleomorphic cells	pleomorphic cells	pleomorphic rods
Cell arrangement	single and paired cells	single, paired cells and long chains	single and paired cells	single and paired cells	paired cells and long chains	single and paired cells	single and paired cells	paired chains
Chains	−	+	−	−	+	−	−	+
Motile	−	−	−	−	+	−	−	+
Cell size; length and width (*μ*m)	2–5 × 1	3–7.5 × 1	1–4 × 1	1–4 × 1	2–5 × 1	3–7.5 × 1	2–7.5 × 1.75	2–7.5 × 1
Oxidase	+	−	+	−	+	−	−	−
Catalase	−	−	−	−	−	−	+	−

*Growth at *37°*C, pH 7.2*								
0% NaCl	−	−	−	−	−	−	−	−
2% NaCl	+	−	−	−	+	−	+	+
5% NaCl	+	−	−	−	+	−	+	+
8% NaCl	+	+	−	−	+	+	+	+
10% NaCl	+	+	+	+	+	+	+	+
15% NaCl	+	+	+	+	+	+	+	+
25% NaCl	+	+	+	+	+	+	+	+
30% NaCl	−	−	−	−	−	−	−	−

*Growth at *37°*C*								
pH 4.5	−	−	−	−	−	−	−	−
pH 6	+	+	+	+	+	+	+	+
pH 7	+	+	+	+	+	+	+	+
pH 7.5	+	+	+	+	+	+	+	+
pH 8	−	−	−	−	−	−	−	−

*API 20E:*								
Hydrolysis of:								
ONPG	−	−	−	−	−	−	−	−
Arginine dihydrolase	+	+	+	+	+	+	+	+
Lysine decarboxylase	+	+	+	+	+	+	+	+
Ornithine decarboxylase	+	+	+	+	+	+	+	+
Citrate utilization	−	−	−	−	−	−	−	+
H_2_S production	−	−	−	−	−	−	−	−
Urease	+	+	+	+	+	+	+	+
Tryptophan deaminase	−	−	−	−	−	−	−	−
Indol production	−	−	−	−	−	−	−	−
Voges-Proskauer	+	+	+	−	−	−	−	−
Gelatinase activity	−	−	−	−	−	−	−	−
Fermentation/oxidation:								
D-glucose	−	−	−	−	−	−	−	−
D-mannitol	−	−	−	−	−	−	−	−
Inositol	−	−	−	−	−	−	−	−
D-sorbitol	−	−	−	−	−	−	−	−
L-rhamnose	−	−	−	−	−	−	−	−
D-sucrose	−	−	−	−	−	−	−	−
D-melibiose	−	−	−	−	−	−	−	−
Amygdalin	−	−	−	−	−	−	−	−
L-arabinose	−	−	−	−	−	−	−	−

*API 20NE:*								
NO_3_ reduction	−	−	−	−	−	−	+	+
NO_2_ reduction	−	−	−	−	−	−	−	−
Hydrolysis of:								
Aesculin	−	−	−	−	−	−	−	−
PNPG	−	−	−	−	−	−	−	−
Assimilation of:								
D-mannose	−	−	−	−	−	−	−	−
N-acetyl-glucosamine	+	−	−	−	−	−	−	+
D-maltose	+	−	−	−	−	−	−	−
Potassium gluconate	−	−	−	−	−	−	−	−
Capric acid	−	−	−	−	−	−	−	−
Adipic acid	−	−	−	−	−	−	−	−
Malic acid	+	−	−	−	−	−	−	+
Phenylacetic acid	−	−	−	−	−	−	−	−

Sampling sites (S)/depth (cm)/ characteristics	604 S6/0	6′02S6/0	419 S4/10	402 S4/0	605 S6/0	2019 S2/0	214 S2/10	403 S4/0

Taxonomical status	*Halomonas* sp.	*Halomonas* sp.	*Halomonas* sp.	*Halomonas* sp.	*Halomonas* sp.	*Halomonas* sp.	*Halomonas* sp.	*Halomonas* sp.
Colonial morphology	Circular	Circular	Circular	Circular	irregular and spreading	Circular	Circular	irregular and spreading
Colony size	1 mm convex	1-2 mm convex	1-2 mm convex	1-2 mm convex	1-2 mm convex	1 mm convex	1 mm flat	3 mm convex
Colony density	transparent glistening	opaque matt	opaque matt	opaque matt	translucent glistening	opaque matt	opaque matt	translucent glistening
Pigmentation	cream	white	cream	white	white	white	cream	white
Cell shape	pleomorphic cells	pleomorphic cells	pleomorphic rods	pleomorphic cells	pleomorphic cells	pleomorphic cells	pleomorphic cells	pleomorphic cells
Cell arrangement	single and paired cells	single and paired cells	long chains	single and paired cells	single and paired cells	single and paired cells	single and paired cells	single and paired cells
Chains	−	−	+	+	+	+	−	+
Motile	−	−	−	+	−	+	+	+
Cell size; length and width (*μ*m)	2–5 × 1	3–7.5 × 1.5	3–7.5 × 1	2–7.5 × 1	2–7.5 × 1	2–5 × 1	2–5 × 1	2–7.5 × 1
Oxidase	−	−	−	−	−	−	−	−
Catalase	−	−	−	−	−	−	−	−

*Growth at *37°*C, pH 7.2*								
0% NaCl	−	−	−	−	−	−	−	−
2% NaCl	−	−	−	+	+	+	+	+
5% NaCl	−	−	−	+	+	+	+	+
8% NaCl	+	+	+	+	+	+	+	+
10% NaCl	+	+	+	+	+	+	+	+
15% NaCl	+	+	+	+	+	+	+	+
25% NaCl	+	+	+	+	+	+	+	+
30% NaCl	−	−	−	−	−	−	−	−

*Growth at *37°*C*								
pH 4.5	−	−	−	−	−	−	−	−
pH 6	+	+	+	+	+	+	+	+
pH 7	+	+	+	+	+	+	+	+
pH 7.5	+	+	+	+	+	+	+	+
pH 8	−	−	−	−	−	−	−	−

*API 20E:*								
Hydrolysis of:								
ONPG	−	−	−	−	−	−	−	−
Arginine dihydrolase	+	+	+	+	+	+	+	+
Lysine decarboxylase	+	+	+	+	+	+	+	+
Ornithine decarboxylase	+	+	+	+	+	+	+	+
Citrate utilization	−	−	−	+	+	−	−	+
H_2_S production	−	−	−	−	−	−	−	−
Urease	+	+	+	+	+	+	+	+
Tryptophan deaminase	−	−	−	−	−	−	−	−
Indol production	−	−	−	−	−	−	−	−
Voges-Proskauer	−	−	−	−	−	−	−	−
Gelatinase activity	−	−	−	−	−	−	−	−
Fermentation/oxidation:								
D-glucose	−	−	−	−	−	−	−	+
D-mannitol	−	−	−	−	−	−	−	−
Inositol	−	−	−	−	−	−	−	−
D-sorbitol	−	−	−	−	−	−	−	−
L-rhamnose	−	−	−	−	−	−	−	−
D-sucrose	−	−	−	−	−	−	−	−
D-melibiose	−	−	−	−	−	−	−	−
Amygdalin	−	−	−	−	−	−	−	−
L-arabinose	−	−	−	−	−	−	−	−

*API 20NE:*								
NO_3_ reduction	−	−	−	+	+	+	−	+
NO_2_ reduction	−	−	−	−	−	−	−	−
Hydrolysis of:								
Aesculin	−	−	−	−	−	−	−	−
PNPG	−	−	−	−	−	−	−	−
Assimilation of:								
D-mannose	−	−	−	−	−	−	−	−
N-acetyl-glucosamine	−	−	−	+	−	−	−	−
D-maltose	−	−	−	−	−	−	−	−
Potassium gluconate	−	−	−	−	−	−	−	−
Capric acid	−	−	−	−	−	−	−	−
Adipic acid	−	−	−	−	−	−	−	−
Malic acid	−	−	−	−	−	−	−	−
Phenylacetic acid	−	−	−	−	−	−	−	−

Sampling sites (S)/depth (cm)/ characteristics	139 S1/30	2015 S2/0	1′010S1/0	2230 S2/20	101 S1/0	1′115S1/10	1′213S1/20	108 S1/0

Taxonomical status	*Halomonas* sp.	*Halomonas* sp.	*Halomonas* sp.	*Halomonas* sp.	*Halomonas* sp.	*Halomonas* sp.	*Halomonas* sp.	*Halomonas* sp.
Colonial morphology	Irregular and spreading	Circular	Circular	Circular	Circular	irregular and spreading	Circular	irregular and spreading
Colony size	1-2 mm convex	1 mm convex	1-2 mm convex	1 mm convex	1 mm convex	1-2 mm convex	0.3 mm slightly raised	1-2 mm convex
Colony density	translucent glistening	opaque matt	translucent glistening	opaque matt	translucent glistening	translucent glistening	translucent matt	translucent glistening
Pigmentation	white	white	white	cream	white	white	white	white
Cell shape	pleomorphic cells	Pleomorphic rods	pleomorphic cells	pleomorphic cells	pleomorphic rods	pleomorphic rods	pleomorphic cells	pleomorphic cells
Cell arrangement	single and paired cells	long chains	single and paired cells	single and paired cells	paired cells	single and paired cells	single and paired cells	single and paired cells
Chains	−	+	−	+	+	+	−	+
Motile	−	+	−	+	+	+	−	−
Cell size; length and width (*μ*m)	2–7.5 × 1	2–5 × 1	2–7.5 × 1.75	2–5 × 1	2–7.5 × 1	2–7.5 × 1.5	2–7.5 × 1	2–7.5 × 1
Oxidase	−	+	−	−	−	−	−	−
Catalase	−	−	−	−	−	−	+	−

*Growth at *37°*C, pH 7.2*								
0% NaCl	−	−	−	−	−	−	−	−
2% NaCl	+	+	+	+	+	+	−	+
5% NaCl	+	+	+	+	+	+	+	+
8% NaCl	+	+	+	+	+	+	+	+
10% NaCl	+	+	+	+	+	+	+	+
15% NaCl	+	+	+	+	+	+	+	+
25% NaCl	+	+	+	+	+	−	+	+
30% NaCl	−	−	−	−	−	−	−	−

*Growth at *37°*C*								
pH 4.5	−	−	−	−	−	−	−	−
pH 6	+	+	+	+	+	+	+	+
pH 7	+	+	+	+	+	+	+	+
pH 7.5	+	+	+	+	+	+	+	+
pH 8	−	−	−	−	−	−	−	−

*API 20E:*								
Hydrolysis of:								
ONPG	−	−	−	−	−	−	−	−
Arginine dihydrolase	+	+	+	+	+	+	+	+
Lysine decarboxylase	+	+	+	+	+	+	+	+
Ornithine decarboxylase	+	+	+	+	+	+	+	+
Citrate utilization	−	−	−	−	+	−	−	−
H_2_S production	−	−	−	−	−	−	−	−
Urease	+	+	+	+	+	−	+	+
Tryptophan deaminase	−	−	−	−	−	−	−	−
Indol production	−	−	−	−	−	−	−	−
Voges-Proskauer	−	−	−	−	−	−	−	−
Gelatinase activity	−	−	−	−	−	−	−	−
Fermentation/oxidation:								
D-glucose	−	−	−	−	−	−	−	−
D-mannitol	−	−	−	−	−	−	−	−
Inositol	−	−	−	−	−	−	−	−
D-sorbitol	−	−	−	−	−	−	−	−
L-rhamnose	−	−	−	−	−	−	−	−
D-sucrose	−	−	−	−	−	−	−	−
D-melibiose	−	−	−	−	−	−	−	−
Amygdalin	−	−	−	−	−	−	−	−
L-arabinose	−	−	−	−	−	−	−	−

*API 20NE:*								
NO_3_ reduction	+	−	+	−	+	+	+	+
NO_2_ reduction	−	+	−	−	−	−	−	−
Hydrolysis of:								
Aesculin	−	−	−	−	−	−	−	−
PNPG	−	−	−	−	−	−	−	−
Assimilation of:								
D-mannose	−	−	−	−	−	−	−	−
N-acetyl-glucosamine	−	−	−	−	−	−	−	−
D-maltose	−	−	−	−	−	−	−	−
Potassium gluconate	−	−	−	−	−	−	−	−
Capric acid	−	−	−	−	−	−	−	−
Adipic acid	−	−	−	−	−	−	−	−
Malic acid	−	−	−	−	−	−	−	−
Phenylacetic acid	−	−	−	−	−	−	−	−

Sampling sites (S)/depth (cm)/ characteristics	137 S1/30	103 S1/0	3′020S3/0	326 S3/20	304 S3/0	3′09S3/0	426 S4/20	3216 S3/20

Taxonomical status	*Halomonas* sp.	*Halomonas* sp.	*Salicola* sp.	*Salicola* sp.	*Salicola* sp.	*Salicola* sp.	*Salicola* sp.	*Salicola* sp.
Colonial morphology	irregular and spreading	Circular	Circular	Circular	Circular	Circular	irregular and spreading	Circular
Colony size	1-2 mm convex	0.5–1 mm convex	0.3 mm flat	0.3 mm convex	0.3 mm flat	0.2-0.3 mm flat	1 mm flat	0.2-0.3 mm slightly raised
Colony density	translucent glistening	translucent matt	opaque matt	translucent matt	opaque matt	transparent matt	opaque matt	translucent matt
Pigmentation	white	white	yellowish- cream	white	transparent white	transparent- white	transparent- white	transparent- white
Cell shape	pleomorphic cells	pleomorphic cells	pleomorphic rods	pleomorphic cells	pleomorphic rods	pleomorphic cells	pleomorphic cells	pleomorphic cells
Cell arrangement	single and paired cells	single and paired cells	single and paired cells	single and paired cells	single and paired cells	single and paired cells	single and paired cells	single and paired cells
Chains	−	+	−	+	+	−	−	−
Motile	−	+	−	−	−	−	+	−
Cell size; length and width (*μ*m)	2–7.5 × 1	2–7.5 × 1.5	1–3 × 0.5	1–4 × 0.5	1–3 × 0.5	1–3 × 0.5	1–4 × 0.5	1–4 × 0.5
Oxidase	−	−	−	−	−	−	−	−
Catalase	−	−	−	−	−	−	−	−

*Growth at *37°*C, pH 7.2*								
0% NaCl	−	−	−	−	−	−	−	−
2% NaCl	+	+	−	−	−	−	−	−
5% NaCl	+	+	−	−	−	−	+	−
8% NaCl	+	+	−	+	−	−	+	−
10% NaCl	+	+	+	+	+	+	+	+
15% NaCl	+	+	+	+	+	+	+	+
25% NaCl	+	+	+	+	+	+	+	+
30% NaCl	−	−	−	−	−	−	−	−

*Growth at *37°*C*								
pH 4.5	−	−	−	−	−	−	−	−
pH 6	+	+	+	+	+	+	+	+
pH 7	+	+	+	+	+	+	+	+
pH 7.5	+	+	+	+	+	+	+	+
pH 8	−	−	−	−	−	−	−	−

*API 20E:*								
Hydrolysis of:								
ONPG	−	−	−	−	−	−	−	−
Arginine dihydrolase	+	+	+	+	+	+	+	+
Lysine decarboxylase	+	+	+	+	+	+	+	+
Ornithine decarboxylase	+	+	+	+	+	+	+	+
Citrate utilization	−	+	−	−	−	−	−	+
H_2_S production	−	−	−	−	−	−	−	−
Urease	+	+	+	+	+	+	+	+
Tryptophan deaminase	−	−	−	−	−	−	−	−
Indol production	−	−	−	−	−	−	−	−
Voges-Proskauer	−	−	−	−	−	−	+	−
Gelatinase activity	−	−	−	−	−	−	−	−
Fermentation/oxidation:								
D-glucose	−	−	−	−	−	−	−	−
D-mannitol	−	−	−	−	−	−	−	−
Inositol	−	−	−	−	−	−	−	−
D-sorbitol	−	−	−	−	−	−	−	−
L-rhamnose	−	−	−	−	−	−	−	−
D-sucrose	−	−	−	−	−	−	−	−
D-melibiose	−	−	−	−	−	−	−	−
Amygdalin	−	−	+	+	−	−	−	−
L-arabinose	−	−	−	−	−	−	−	−

*API 20NE:*								
NO_3_ reduction	+	+	+	+	−	−	−	−
NO_2_ reduction	−	−	−	−	−	−	−	−
Hydrolysis of:								
Aesculin	−	−	−	−	−	−	−	−
PNPG	−	−	−	−	−	−	−	−
Assimilation of:								
D-mannose	−	−	−	−	−	−	−	−
N-acetyl-glucosamine	−	−	−	−	−	−	−	−
D-maltose	−	−	−	−	−	−	−	−
Potassium gluconate	−	−	−	−	−	−	−	−
Capric acid	−	−	−	−	−	−	−	−
Adipic acid	−	−	−	−	−	−	−	−
Malic acid	−	−	+	+	−	−	−	−
Phenylacetic acid	−	−	−	−	−	−	−	−

Sampling sites (S)/depth (cm)/ characteristics		105 S1/0		2018 S2/0		411 S4/10		4115 S4/10

Taxonomical status		*Marinococcus* sp.		*Pontibacillus* sp.		*Halobacillus* sp.		*Halorubrum* sp.
Colonial morphology		Circular		Circular		Circular		Circular
Colony size		1 mm convex		1 mm flat		1 mm flat		0.3 mm slightly raised
Colony density		opaque matt		opaque matt		opaque matt		opaque matt
Pigmentation		reddish- orange		cream		cream		brick-red
Cell shape		pleomorphic rods		pleomorphic rods		pleomorphic rods		pleomorphic cells
Cell arrangement		single, paired and irregularly clustered cells		long chains		long chains		single and paired cells
Chains		−		+		+		−
Motile		−		+		+		+
Cell size; length and width (*μ*m)		1–5 × 2		1–4 × 1		1–4 × 1		1–4 × 1
Oxidase		−		−		−		−
Catalase		+		−		−		−

*Growth at *37°*C, pH 7.2*								
0% NaCl		−		−		−		−
2% NaCl		−		−		−		−
5% NaCl		+		+		+		−
8% NaCl		+		+		+		+
10% NaCl		+		+		+		+
15% NaCl		+		+		+		+
25% NaCl		+		+		+		+
30% NaCl		−		−		−		−

*Growth at *37°*C*								
pH 4.5		−		−		−		−
pH 6		+		+		+		+
pH 7		+		+		+		+
pH 7.5		+		+		+		+
pH 8		−		−		−		−

*API 20E:*								
Hydrolysis of:								
ONPG		−		−		−		−
Arginine dihydrolase		+		+		+		+
Lysine decarboxylase		+		+		+		+
Ornithine decarboxylase		+		+		+		+
Citrate utilization		+		+		+		+
H_2_S production		−		−		−		−
Urease		+		+		+		+
Tryptophan deaminase		−		−		−		−
Indol production		−		−		−		−
Voges-Proskauer		−		−		−		−
Gelatinase activity		−		−		−		−
Fermentation/oxidation:								
D-glucose		−		−		−		−
D-mannitol		−		−		−		−
Inositol		−		−		−		−
D-sorbitol		−		−		−		−
L-rhamnose		−		−		−		−
D-sucrose		−		−		−		−
D-melibiose		−		−		−		−
Amygdalin		−		−		−		−
L-arabinose		−		−		−		−

*API 20NE:*								
NO_3_ reduction		−		−		−		−
NO_2_ reduction		−		−		−		−
Hydrolysis of:								
Aesculin		−		−		−		−
PNPG		−		−		−		−
Assimilation of:								
D-mannose		−		−		−		−
N-acetyl-glucosamine		−		−		−		−
D-maltose		−		−		−		−
Potassium gluconate		−		−		−		−
Capric acid		−		−		−		−
Adipic acid		−		−		−		−
Malic acid		−		−		−		−
Phenylacetic acid		−		−		−		−
